# Epidemiology of antibiotic consumption and resistance in Mauritius

**DOI:** 10.3389/frabi.2024.1222580

**Published:** 2024-04-16

**Authors:** Lovena Preeyadarshini Veerapa-Mangroo, Harena Rasamoelina-Andriamanivo, Mohammad Iqbal Issack, Eric Cardinale

**Affiliations:** ^1^Surveillance Epidemiologique et Gestion des Alertes (SEGA) One Health network, Indian Ocean Commission, Ebene, Mauritius; ^2^Epidemic Intelligence Unit, Indian Ocean Commission, Ebene, Mauritius; ^3^Central Health Laboratory, Victoria Hospital, Quatre Bornes, Mauritius; ^4^Unité mixte de recherche (UMR) Animal, Santé, Territoires, Risques, Écosystèmes (ASTRE) — Centre de coopération internationale en recherche agronomique pour le développement (CIRAD), Montpellier, France

**Keywords:** antibiotic consumption, human and animal sector, antibiotic resistance, multi-drug resistant organisms, Mauritius, tropical island, epidemiology, defined daily dose

## Abstract

**Introduction:**

This study aims at determining the pattern of antibiotic consumption and resistance in Mauritius, a tropical island in the Indian Ocean.

**Methodology:**

Antibiotic consumption was measured in kilograms of purchased antibiotics and also in defined daily dose (DDD) in different health institutions from 2015 to 2017. Data on antibiotic resistance was collected at the Central Health Laboratory (CHL) at Victoria Hospital and at Jeetoo Hospital Laboratory, where antibiotic sensitivity testing is done for all public health institutions. For this study, *Escherichia coli*, *Klebsiella* species, *Acinetobacter* species, and *Pseudomonas aeruginosa* isolates from blood samples of patients from 2015 to 2023 were included. The resistance rate and prevalence of multi-drug-resistant (MDR) organisms were calculated.

**Results:**

The amount of antibiotics (in kilograms) distributed to the human sector was between 11,000 to 13,000 kg, compared to only 700 to 1,500 kg in the animal sector. The DDD per 1,000 inhabitants per day was 20.9, 22.1, and 21.7 in 2015, 2016, and 2017, respectively, with a greater consumption of WATCH and RESERVE group antibiotics in the private sector. In public health institutions, health centers in the northern region had the highest DDD per 1,000 outpatients per day for beta-lactams penicillins and quinolones. Concerning antibiotic resistance, the proportion of MDR *Acinetobacter baumannii* and *Pseudomonas aeruginosa* has increased from 58% to 74% and from 33% to 45%, respectively, from 2015 to 2023. During the same period, the proportion of *E. coli and K. pneumoniae* isolates sensitive to ceftriaxone decreased from 55% to 39% and from 37% to 22%, respectively, while the proportion of *E. coli*, *Klebsiella pneumoniae*, *Acinetobacter baumannii*, and *Pseudomonas aeruginosa* isolates sensitive to meropenem decreased from 98% to 94%, 83% to 53%, 45% to 28%, and 63% to 47%, respectively.

**Conclusion:**

This study provides valuable insights on antibiotic consumption and resistance in the country and emphasizes the significance of adopting a One Health approach to combat antimicrobial resistance (AMR) effectively. These findings will aid policymakers in formulating targeted strategies to address the challenge of AMR and should be integrated into the National Action Plan on AMR in Mauritius.

## 1 Introduction

The discovery of antibiotics stands as one of the most critical advances in medical history. It has saved countless lives and revolutionized the treatment of infections (Derderian, 2007). However, their efficacy is now being threatened due to the misuse and overuse of antibiotics in humans, animals, and plants. Between 2000 and 2015, the antibiotic consumption rates worldwide increased by 39%, from 11.3 to 15.7 defined daily doses per 1,000 inhabitants per day (DID) (Klein et al., 2021). This trend is expected to continue, and the consumption of antibiotics could double by 2030 (Klein et al., 2021). The same report shows an increase in the consumption of last-resort antibiotics such as carbapenems and polymyxins across the world (Klein et al., 2021). We cannot ignore the implications of this massive selection pressure on bacterial resistance mechanisms. Research reveals that this is causing an acceleration in the said mechanisms, rendering antibiotics ineffective (Oz et al., 2014; Klein et al., 2021). A few examples of such multi-resistance mechanisms include extended spectrum beta-lactamase Enterobacteriaceae (ESBL), carbapenem-resistant Enterobacteriaceae (CRE), and the mcr-1 gene, all of which are rising at an alarming pace globally (Gröndahl-Yli-Hannuksela et al., 2018; Klein et al., 2021; World Health Organization, 2021; Bose et al., 2022). This poses a serious challenge to the treatment of infected individuals, as multi-resistant strains require newer and more expensive antibiotics that are often unaffordable in low- and middle-income countries (Derderian, 2007). This underscores the urgent need for effective strategies in combating the spread of these resistant mechanisms.

In 2015, the World Health Organization (WHO) developed a global action plan on antimicrobial resistance with one of the objectives being to strengthen knowledge through surveillance and research (World Health Organization, 2015). Surveillance remains an essential component to understand the burden of antibiotic consumption and resistance so that evidence-based recommendations specific to the country and local context can be formulated. Globally, research should be continued to understand the causes and mechanisms of antibiotic resistance. This will provide further information on the consumption of antibiotics and resistance of bacteria and shed light on the complex relationship between them (Bakhit et al., 2018; Goosens, 2009; Tamminen et al., 2011; Meyer et al., 2013; Bell et al., 2014; Baditoiu et al., 2017).

The healthcare system in Mauritius consists of both public and private sectors. The public sector is organized into primary, secondary, and tertiary healthcare levels, with a total of 141 health centers, two district hospitals, five regional hospitals, and eight specialized hospitals spread throughout the island (Ministry of Health and Wellness, 2017). The five regional hospitals offer both outpatient and inpatient care. Treatment in the public healthcare system is provided free of charge, including medications and laboratory tests (Ministry of Health and Wellness, 2017). The Pharmacy Board oversees the importation of antibiotics for human and animal use, while the Central Supply Division, a sub-branch of the Pharmacy Board, handles the distribution of antibiotics in the public sector (Market Study, 2020). Mauritius has a Pharmacy Act which stipulates that antibiotics should only be dispensed with a prescription from a registered doctor or veterinarian (Mauritius The Pharmacy Act 1983, 1983). So far, there has been no comprehensive analysis on the antibiotic consumption in Mauritius.

According to the Ministry of Agro-Industry, in 2017, the number of poultry, pigs, small ruminants, cattle, and domestic dogs was estimated at eight million, 20,000, 20,000, 6,000, and 200,000, respectively. The human population was estimated at 1.22 million in 2017 (Ministry of Health and Wellness, 2017).

The Central Health Laboratory at Victoria Hospital and the microbiology laboratory at Jeetoo Hospital conduct antibiotic susceptibility testing (AST) daily on samples received from hospitals or health centers for the human sector. *Klebsiella* strains producing ESBL and NDM-1 enzymes have been identified in isolates from Mauritius (Poirel et al., 2012; Mungloo-Rujubali et al., 2013). Other studies further highlight the growing issue of antibiotic resistance in Mauritius—for instance, between March 2005 and July 2014, the resistance of *Escherichia coli* to cefotaxime among hospitalized patients (*N* = 84 in 2005 and *N* = 183 in 2014) increased from 18% to 46%, and resistance to ciprofloxacin increased from 24% to 58% (Issack et al., 2007; Issack and Manraj, 2012; Issack, 2016). Another study shows that 68% of 214 organisms in an intensive care unit of Mauritius were multi-drug-resistant organisms, with 78% being ESBL-producing Enterobacteriaceae (Nuckchady and Boolaky, 2020). Moreover, a study done in Reunion Island revealed a high prevalence of CRE in patients from Mauritius (Gay et al., 2020). These findings indicate that, like in other parts of the world, Mauritius is also facing the challenge of antibiotic resistance.

Mauritius is a tropical island in the middle of the Indian Ocean (The World Bank in Mauritius, 2022). It is one of the five member countries of the Indian Ocean Commission (IOC) (Reseau SEGA, One Health and Indian Ocean Commission, 2022). The Communicable Disease Control Unit of the Ministry of Health and Wellness of Mauritius collaborates with the SEGA network, “Surveillance Epidemiologique et Gestion des Alertes”, i.e., Epidemiologic Surveillance and Response Unit of the IOC and the French Agricultural Research Centre for International Development in Reunion toward a One Health approach to antibiotic resistance (French Agricultural Research Centre for International Development (CIRAD), 2022; Reseau SEGA, One Health and Indian Ocean Commission, 2022). This study aims to determine, for the first time in Mauritius, the pattern of antibiotic consumption in different sectors and the prevalence of antibiotic resistance by ward type in the regional hospitals of Mauritius. The findings of this study will help to inform policy decisions and guide the development of effective strategies to combat AMR on the island.

## 2 Materials and methods

This study is a retrospective descriptive analysis of the consumption patterns of purchased antibiotics in both human and animal sectors of Mauritius, covering the period from January 2015 to December 2017. Additionally, the study also examines the prevalence of antibiotic resistance in the public human health sector from 2015 to 2023.

Antibiotic consumption data was obtained from the Pharmacy Board and the Ministry of Agro-Industry. The antibiotics distributed to the health centers and hospitals were provided by the CSD, while the antibiotics distributed to the private pharmacies and private farms were obtained *via* private companies. The Ministry of Agro-Industry provided the data on antibiotics used by them. The data were then collected and analyzed on Microsoft Excel.

The total antibiotics distributed to the institutions of the country were calculated in kilograms in both sectors irrespective of the route of administration.

Further analysis on antibiotic consumption was done for the human sector. The antibiotics were coded according to the Anatomical Therapeutic and Chemical classification on the WHO Collaborating Centre for Drugs Statistics Methodology website (World Health Organization Collaborating Centre for Drug Statistics Methodology, 2023). The defined daily dose (DDD) indicator was used to measure systemic antibiotics for human consumption (World Health Organization Collaborating Centre for Drug Statistics Methodology, 2023). Only oral and parenteral antibiotics were included, and all topical and drops were excluded in the calculation of the DDD. The DID was determined for antibiotic consumption in both private and public sectors of the population. The antibiotics were classified according to the AWARE classification, and the proportion of antibiotics used in the ACCESS, WATCH, and RESERVE groups was calculated (World Health Organization, 2019b).

Moreover, in the public sector, additional indicators were calculated, such as the DDD per 1,000 outpatients per day and DDD per 1,000 admissions per day. The number of outpatients and admissions per institution was obtained from the Mauritius Health Records department (Ministry of Health and Wellness, 2016, 2017). The DDD per 1,000 outpatients per day and DDD per 1,000 admissions per day were calculated for the health centers, regional hospitals, specialized hospitals, and district hospitals. A point prevalence survey on antibiotic use was carried out in three regional hospitals in 2018 to 2019, and it was found that more than 80% of the admitted patients were given parenteral antibiotics compared to oral (Veerapa-Mangroo et al., 2023). Hence, oral antibiotics used at the regional hospitals were allocated to the outpatient department, while antibiotics administered *via* the parenteral route were allocated to the inpatients.

Data on antibiotic resistance were collected at the Central Health Laboratory (CHL) at Victoria Hospital and at Dr A.G. Jeetoo Hospital, where AST is done for all public institutions all over the island by the Kirby Bauer disk diffusion method and interpreted according to the Clinical and Laboratory Standards Institute (CLSI) breakpoints (Clinical and Laboratory Standards Institute, 2022). For this study, *Escherichia coli*, K*lebsiella* species, *Acinetobacter* species, and *Pseudomonas aeruginosa* isolates from blood samples of patients admitted in regional hospitals were included. The blood samples were sent by the treating doctors and often formed part of the standard patient care. The data were collected from January to December in 2015, 2016, 2017, 2021, and January to May in 2023. Multi-drug-resistant (MDR) organisms were those that developed acquired resistance to three or more classes of antibiotics. They were then analyzed on Epi Info and Microsoft Excel. All duplicates were removed prior to the analysis of data. The Ethics Committee of the Ministry of Health and Wellness gave approval for this study (Ref MHC/CT/NETH/VMLP).

## 3 Results

### 3.1 Antibiotic consumption

#### 3.1.1 Total antibiotic consumption in human and animal sectors

The total antibiotic consumption ranged from 12,000 to 14,000 kg from 2015 to 2017, as illustrated in **Table 1**. In the human sector, approximately 11,000 to 13,000 kg of antibiotics were used to treat the population of Mauritius which is approximately 1.22 million inhabitants (Ministry of Health and Wellness, 2017). For the animal sector, 700 to 1,500 kg of antibiotics was used to treat the different animal species which were estimated by the Ministry of Agro-Industry to be approximately 8 million poultry, 20,000 pigs, 20,000 small ruminants, 6,000 cattle, and 200,000 domestic dogs in 2017. In 2017, 7,607 kg of penicillins, 1,180 kg of quinolones, and 765 kg of sulphonamides were consumed in all sectors. In the same year, 339 and 80 kg of tetracyclines and 142 and 623 kg of sulfonamides were used in the animal and human sectors, respectively. The amount of antibiotics consumed by the animal sector has decreased from 1,527 to 700 kg from 2015 to 2017 (**Table 1**).

**Table 1:** Antibiotic consumption (in kilograms) in the human and animal sectors of Mauritius from 2015 to 2017.

	Human			Animal			Total		
By year	2015	2016	2017	2015	2016	2017	2015	2016	2017
Class of antibiotics									
Penicillins	8,177	8,543	7,474	91	102	133	8,268	8,645	7,607
Quinolones	1,255	1,469	1,136	40	37	44	1,295	1,506	1,180
Sulfonamides	581	629	623	211	126	142	792	755	765
Nitroimidazoles	743	910	755	1	1	2	744	911	757
Macrolides	607	741	687	31	21	9	638	762	696
Cephalosporins	660	644	698	4	4	4	664	648	702
Carbapenems	36	40	32	0	0	0	36	40	32
Tetracyclines	86	74	80	1,110	469	339	1,196	543	419
Aminoglycosides	66	26	18	38	59	27	104	85	45
Glycopeptides	8	13	9	0	0	0	8	13	9
Lincosamides	27	36	32	0	0	0	27	36	32
Amphenicol	9	5	5	0	0	0	9	5	5
Steroid antibacterial	5	2	2	0	0	0	5	2	2
Streptogramins	0	1	0	0	0	0	0	1	0
Polymyxin	0	0	0	1	1	0	1	1	0
Total (kg)	12,234	13,098	11,517	1,527	820	700	13,761	13,918	12,217

#### 3.1.2 Antibiotic consumption in the human sector

The DID was 20.9, 22.1, and 21.7 in 2015, 2016, and 2017, respectively (**Table 2**). In the public sector, the consumption of beta-lactam antibacterial penicillins and quinolones fell from 2015 to 2017, while that of tetracyclines, sulfonamides, and cephalosporins had a general increase. In the private sector, the DID of macrolides, sulfonamides and cephalosporins increased from 2015 to 2017. The DID of tetracyclines, macrolides, lincosamides, and streptogramins group, and cephalosporins in the private sector was greater than that in the public sector (**Table 2**).

**Table 2:** Defined daily dose per 1,000 inhabitants per day (DID) by ATC classification of antibiotics in Mauritius from 2015 to 2017.

ATC classification of antibiotics	Public			Private			Total		
	2015	2016	2017	2015	2016	2017	2015	2016	2017
J01C beta-lactam antibacterials, penicillins	9.50	9.42	8.17	2.48	2.46	3.81	11.98	11.88	11.98
J01CA penicillins with extended spectrum	7.20	6.95	5.63	1.05	1.05	1.24	8.25	8.00	6.87
J01CR combinations of penicillins	1.14	1.37	1.32	1.37	1.24	2.54	2.51	2.61	3.86
J01CF beta-lactamase-resistant penicillins	1.16	1.10	1.22	0.06	0.17	0.03	1.22	1.27	1.25
J01M quinolone antibacterials (J01 MA fluoroquinolones)	2.19	1.85	1.77	0.87	1.81	1.02	3.06	3.66	2.79
J01F macrolides, lincosamides, and streptogramins	1.11	1.31	1.21	1.23	1.83	1.91	2.34	3.14	3.12
J01A tetracyclines	0.28	0.30	0.36	1.04	0.94	1.07	1.32	1.24	1.43
J01E sulfonamides and trimethoprim	0.29	0.30	0.31	0.01	0.01	0.04	0.30	0.31	0.35
J01D other beta-lactam antibacterials	0.30	0.29	0.31	1.02	1.10	1.46	1.32	1.39	1.77
J01DB/C/D other beta-lactam antibacterials–cephalosporins^a^	0.28	0.27	0.30	1.01	1.09	1.45	1.29	1.36	1.75
J01DH other beta-lactam antibacterials–carbapenems	0.02	0.02	0.01	0.01	0.01	0.01	0.03	0.03	0.02
J01G aminoglycosides	0.04	0.04	0.05	0.15	0.03	0.06	0.14	0.07	0.11
J01X other antibacterials	0.10	0.20	0.10	0.24	0.20	0.03	0.30	0.40	0.10
Total DID	13.81	13.71	12.28	7.04	8.38	9.40	20.85	22.09	21.68

a Only first-, second-, and third-generation cephalosporins.

**Figure 1:**
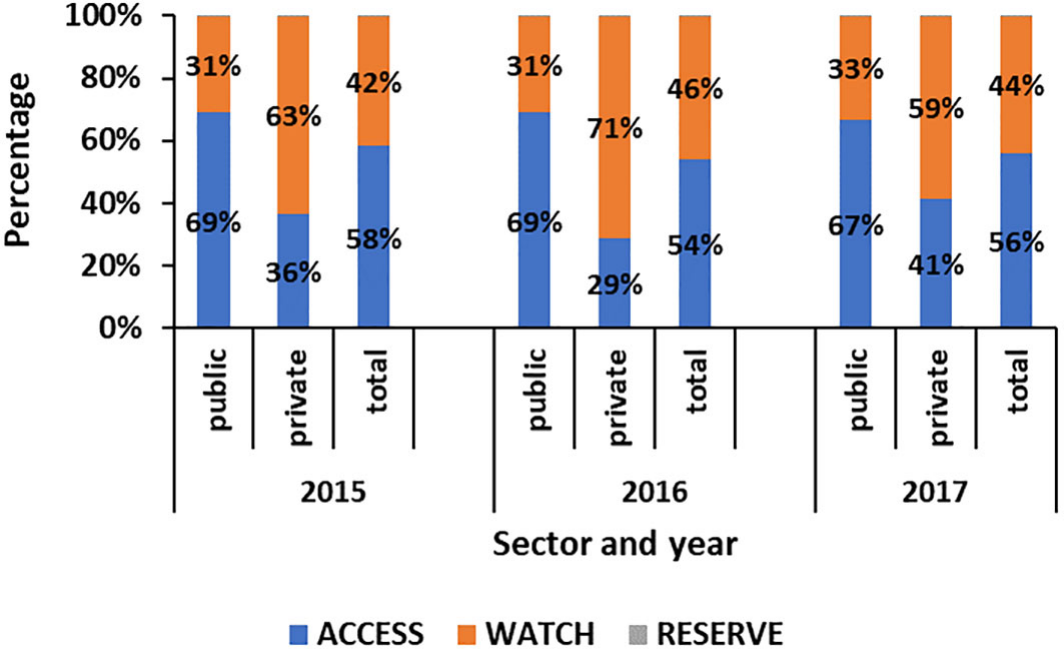
Proportion of antibiotics classified by the AWARE category in the public and private sectors in Mauritius from 2015 to 2017.

**Figure 1** shows the proportion of antibiotics classified according to the AWARE group of antibiotics. It shows that, in the public health sector, the proportion of ACCESS and WATCH antibiotics used were 67%–69% and 31%–33%, respectively. On the other hand, in the private health sector, a higher range of 59%–71% of WATCH antibiotics were used.

**Figure 2:**
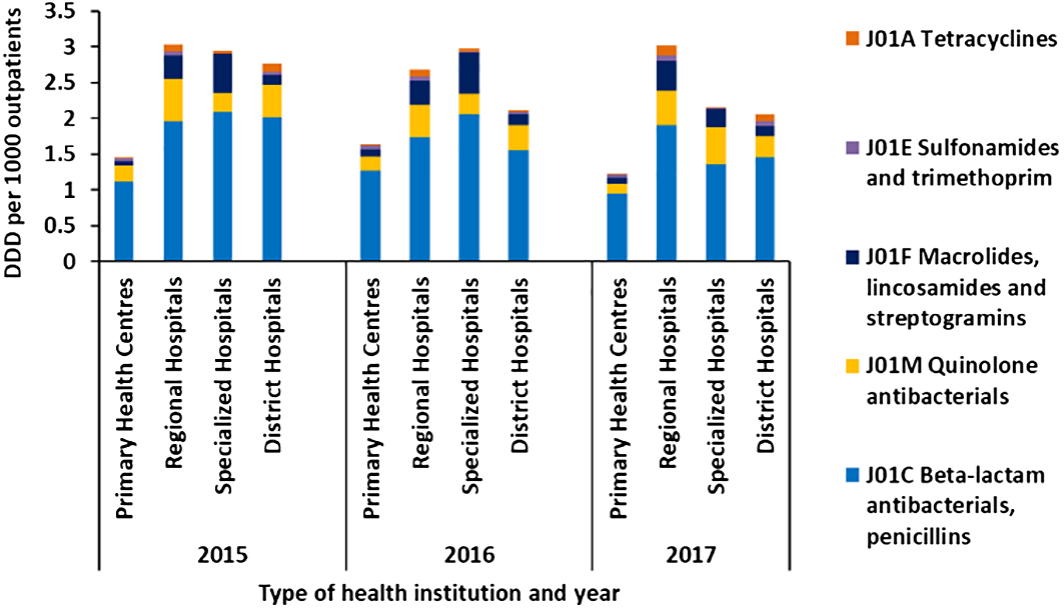
Average defined daily dose per 1,000 outpatients per day by class of antibiotics administered *via* oral route in primary health centers, regional hospitals, specialized hospitals, and district hospitals in Mauritius from 2015 to 2017.

Among the outpatients, penicillins were the most commonly used antibiotic in all institutions, followed by quinolones and macrolides, lincosamides, and streptogramins group, as shown in **Figure 2**.

**Figure 3:**
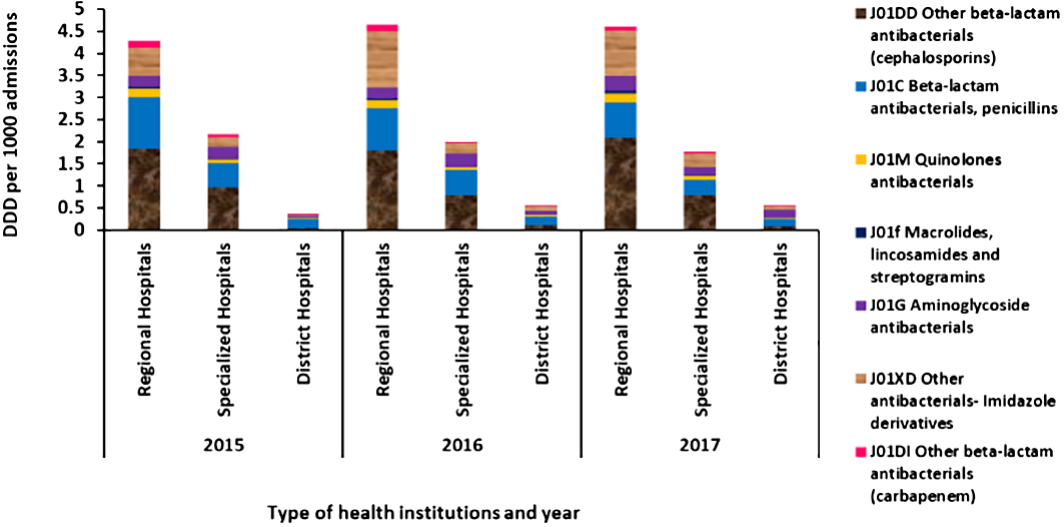
Average defined daily dose per 1,000 admissions per day for antibiotics administered *via* parenteral route in regional hospitals, specialized hospitals, and district hospitals in Mauritius from 2015 to 2017.

Among the inpatients, as shown in **Figure 3**, the other beta-lactam antibacterial cephalosporins were the most frequently used antibiotic followed by penicillins and imidazole derivatives. The DDD per 1,000 admissions per day of the carbapenem group was less than 0.01 in all institutions (shown in **Figure 3**), and no parenteral antibiotics were given in primary healthcare centers.

Among the 25 primary health centers with the highest DDD per 1,000 outpatients per day for penicillins in 2017, 15 health centers were found in the northern regions of the island, five health centers in the southwest region of Mauritius, and five health centers were distributed in the east and southeast of the island. Among the 25 primary health centers with the highest DDD per 1,000 outpatients per day for fluoroquinolones in 2017, a total of 16 centers were found in the northern regions of the country, four health centers in the southwest region of Mauritius, and five health centers in the east and southeast of the island. The primary health centers in the northern regions had the highest DDD per 1,000 outpatients per day for beta-lactams penicillins and quinolones.

### 3.2 Antibiotic resistance in the hospitals of Mauritius

**Table 3** shows that the proportion of *E. coli*, *Klebsiella* species, and *Acinetobacter* isolates sensitive to cotrimoxazole, co-amoxiclav, ciprofloxacin, and ceftriaxone was below 55%. Moreover, the proportion of the above-mentioned isolates susceptible to meropenem, ceftriaxone, and ciprofloxacin was seen to decrease from 2015 to 2023. The proportion of *E. coli*, *Klebsiella pneumoniae*, *Acinetobacter baumannii*, and *Pseudomonas aeruginosa* sensitive to meropenem decreased from 2015 to 2023, from 98% to 94%, 83% to 53%, 45% to 28%, and 63% to 47%, respectively. From 2015 to 2023, the proportion of *E. coli* and *K. pneumoniae* isolates sensitive to ceftriaxone decreased from 55% to 39% and from 37% to 22%, respectively.

**Table 3:** Proportion of *E. coli*, *Klebsiella* species, and *Acinetobacter* specimens sensitive to the listed antibiotics isolated in blood cultures collected from regional hospitals in Mauritius from 2015 to 2023.

	Year (number of samples)	Cotrimoxazole	Coamoxiclav	Ciprofloxacin	Gentamycin	Piperacillin/tazobactam	Meropenem	Ceftriaxone	Colistin
*Escherichia coli*	2015 (*n* = 248)	48%	42%	40%	68%	88%	98%	55%	100%
	2016 (*n* = 277)	52%	40%	41%	61%	85%	96%	48%	100%
	2017 (*n* = 347)	49%	40%	48%	64%	82%	96%	46%	100%
	2021 (*n* = 317)^a^	48%	-	49%	–	–	97%	45%	98%
	2023 (*n* = 153)^a,b^	56%	28%	33%	74%	–	94%	39%	99%
*Klebsiella pneumoniae*	2015 (*n* = 263)	44%	35%	42%	48%	68%	83%	37%	100%
	2016 (*n* = 207)	38%	23%	32%	42%	59%	82%	26%	99%
	2017 (*n* = 243)	47%	38%	50%	59%	72%	87%	38%	100%
	2021 (*n* = 245)^a^	35%	–	42%	–	–	65%	27%	93%
	2023 (*n* = 103)^a,b^	32%	23%	30%	45%	–	53%	22%	100%
*Acinetobacter baumannii*	2015 (*n* = 154)	46%	–	32%	36%	35%	45%	–	99%
	2016 (*n* = 194)	46%	–	35%	39%	35%	47%	–	99%
	2017 (*n* = 190)	43%	–	38%	41%	41%	47%	–	100%
	2021 (*n* = 258)^a^	–	–	–	28%	–	25%	–	97%
	2023 (*n* = 99)^a,b^	34%	–	23%	29%	–	28%	–	98%
*Pseudomonas aeruginosa*	2015 (*n* = 63)	–	–	66%	56%	78%	63%	–	100%
	2016 (*n* = 89)	–	–	52%	52%	67%	52%	–	99%
	2017 (*n* = 82)^a^	–	–	64%	67%	72%	67%	–	99%
	2023 (*n* = 55)^a,b^	–	–	51%	51%	55%	47%	–	96%

a According to CLSI 2017, the AST findings for colistin should be reported as resistant or intermediate. However, in this study, for comparison purposes, the intermediate isolates for colistin were labeled as sensitive.

b Data for 2023 are from January to May 2023 compared to data collected from the years 2015, 2016, 2017, and 2021, where data are from January to December in five regional hospitals.

No seasonal trend was observed in the trend of ABR in Mauritius from January to December.

Additionally, it was found that more than 50% of the meropenem-resistant species were isolated in the intensive care units of the regional hospitals.

The MDR *E. coli* represented 35%, 40%, 41%, and 41% of the total *E. coli* isolates in 2015, 2016, 2017, and 2023, respectively. The MDR *Klebsiella* species represented 58%, 62%, 46%, and 60% of the total *Klebsiella* isolates in 2015, 2016, 2017, and 2023, respectively. The MDR *Acinetobacter* represented 58%, 53%, 56%, and 74% of the total *Acinetobacter* isolates in 2015, 2016, 2017, and 2023, respectively. The MDR *Pseudomonas aeruginosa* represented 33%, 27%, 27%, and 45% of the total *Pseudomonas aeruginosa* isolates in 2015, 2016, 2017, and 2023, respectively.

**Figure 4:**
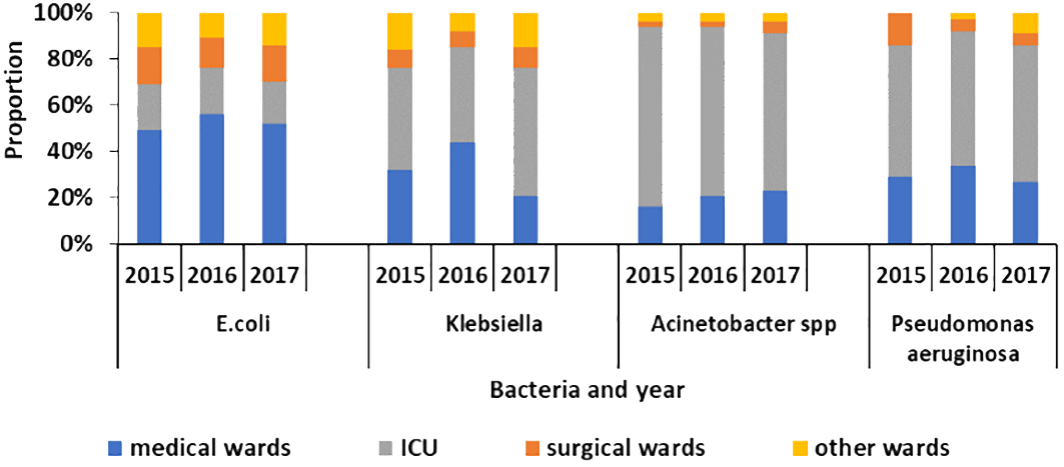
Proportion of multi-drug-resistant *E. coli*, *Klebsiella*, *Acinetobacter* species, and *Pseudomonas aeruginosa* in different ward types in the hospitals of Mauritius from 2015 to 2017.

About 40%–50% of the MDR *Klebsiella*, 60%–80% of MDR *Acinetobacter*, and 50%–60% of *Pseudomonas aeruginosa* were identified in the ICU of the regional hospitals of Mauritius as illustrated in **Figure 4**.

## 4 Discussion

This study provides vital data on the consumption of antibiotics in the human and animal sectors along with antibiotic resistance in the public health institutions of Mauritius from 2015 to 2017. While studies on antibiotic use have been done before in the country, antibiotic consumption is being explored for the first time in Mauritius.

The results provide insights into the Mauritian context, where the majority of antibiotic selection pressure is concentrated in human healthcare institutions. The data indicates that between 2015 to 2017, approximately 11,000 to 13,000 kg of antibiotics was used in the human sector, which serves a population of 1.22 million. In contrast, a smaller amount of 700 to 1,500 kg of antibiotics was used in the animal sector, which consists a population estimate of eight million poultry, 20,000 pigs, 20,000 small ruminants, 6,000 cattle, and 200,000 domestic dogs. This higher consumption of antibiotics in the human sector in Mauritius differs from that of several countries where antimicrobials in the veterinary sector largely exceed that used in human medicine (Moulin et al., 2008; Laxminarayan, 2021). In France, 760 t of antibiotics was used in the human sector, while 1,320 t was used in animals in 2005 (Moulin et al., 2008). While the population correction unit and the DDD are important indicators for monitoring antibiotic consumption in human and animal health, respectively, illustrating the data in kilograms or tonnes allows a comparison of antibiotic consumption in the two sectors (Ferreira, 2017). The findings underline the importance of implementing a One Health national surveillance system to gain a comprehensive understanding of the local context. Establishing an antibiotic stewardship program for human medicine is therefore an effective means of reducing antibiotic consumption and resistance in the country.

The data on antibiotic consumption in the animal sector also shows a decrease from 1,527 to 700 kg from 2015 to 2017. These figures suggest that during the writing of the NAP on AMR in 2016, measures had already been taken by the Ministry of Agro-Industry of Mauritius to decrease antibiotic consumption. Moreover, it is important to note that colistin, an antibiotic in the RESERVE category, is sparingly used in the animal sector.

For the human sector, the consumption of antibiotics in terms of DID in Mauritius for both public and private sectors was 20.9 in 2015, 22.1 in 2016, and 21.7 in 2017. The results suggest that while there is room for improvement, the DID in Mauritius is not excessively high. Among the countries in the European region, the DID in Mauritius is approximately the same as in the United Kingdom (DID 20.1) but lower than in several countries such as France (DID 29.9), Greece (DID 36.1), and Italy (DID 27.5) (European Centre for Disease Prevention and Control, 2018). In the African region, the DID in Mauritius is lower than that of United Republic of Tanzania (DID 27.3) but higher than Burkina Faso (DID 13.8) (World Health Organization, 2019a). In Asia, the DID in India (DID 16) in 2012 and in China (DID 17.4) in 2015 was surprisingly lower compared to that of Mauritius (Farooqui et al., 2018; Yan et al., 2020). However, it is important to interpret the data with caution as the DID reflects the national average, and the population of Mauritius has greater access to free healthcare compared to several countries such as India and China. Data from subsequent years have to be collected to determine a proper trend of DID in Mauritius.

Another important point is the higher DID of other beta-lactams (cephalosporins, carbapenems, and monobactams) in India (DID 2.6) in 2012 and Europe (DID 2.0) in 2017 compared to that of Mauritius (DID 1.8) in 2017 (European Centre for Disease Prevention and Control, 2018; Farooqui et al., 2018). Monitoring the consumption of the WATCH and RESERVE group of antibiotics is crucial to be able to preserve them (World Health Organization, 2019b). In Mauritius, the higher consumption of the WATCH antibiotics in the private sector compared to that of the public sector is of concern (World Health Organization, 2019b). The greater DID of antibiotics in the WATCH category in the private sector was comparable to the findings observed in India where a high proportion of 54.8% of WATCH antibiotics and 27% of ACCESS antibiotics was recorded in 2019 (Koya et al., 2022)—such as the macrolides, lincosamides, and streptogramins group, tetracyclines and cephalosporins were higher in the private sector than in the public sector. These findings are important, especially as the public sector caters to approximately 73% of the healthcare needs of the population, according to a report on the pharmaceutical industry in Mauritius (Market Study, 2020). Hence, the greater proportion of WATCH and RESERVE category of antibiotics is being used to treat only 27% of patients in the private sector (World Health Organization, 2019b). An explanation for this might be the prescribing pattern of the private doctor who wants to meet their patients’ expectations (Fletcher-Lartey et al., 2016). Though a Pharmacy Act exists, its implementation in the country should also be properly monitored to avoid over-the-counter sales in private pharmacies (Mauritius The Pharmacy Act 1983, 1983). To date, in Mauritius, most efforts in tackling AMR have been concentrated in public hospitals. The results show that the private sector should be considered as well during the awareness programs on AMR, and an antibiotic use survey should be carried out regularly to obtain more information on the prescribing practices. This shift to the WATCH and RESERVE category of antibiotics has been observed in other countries as well and might be due to an increasing number of pathogens being resistant to the ACCESS group of antibiotics (Klein et al., 2021).

The health centers having the highest DDD per 1,000 outpatients per day for penicillins and quinolones, the most commonly used antibiotics, are in the northern regions of the country. Concerning antibiotic resistance data, 60% of MDR *Klebsiella pneumoniae* and 62% of MDR *Acinetobacter baumannii* were found in the hospital situated in the north of the island. Possible reasons include the prescribing pattern of the doctors, the patients requesting more antibiotics, or a higher number of infections in these regions.

Additional studies need to be done to determine the cause of this higher antibiotic consumption in those regions. Mauritius, like all countries, has a limited budget and limited skilled human resources. Therefore, using this approach to identify the health centers and corresponding regions with the top DDD is important for national monitoring. It allows the continuous monitoring of antibiotic consumption at the national level and the rapid identification of the health centers or region with the highest DDD. Consequently, appropriate measures such as awareness campaigns can be initiated in the identified regions for both doctors and patients to reduce the overuse and misuse of antibiotics. It is therefore a key strategy in the country to use the data from the pharmacy board and attendances from the health records to monitor and evaluate the progress of antibiotic consumption on the island (Ministry of Health and Wellness, 2016, 2017).

In all institutions of the public sector, the DDD per 1,000 outpatients per day showed a high value for penicillins, followed by quinolones and macrolides, lincosamides, and streptogramins group of antibiotics. Patients with severe symptoms, diseases, and complications have a higher tendency to go to the outpatient departments of regional and specialized hospitals, accounting for the higher DDD per 1,000 outpatients and proportion of macrolides and tetracyclines consumed than in the primary healthcare level.

Concerning the DDD per 1,000 admissions per day, the other beta-lactam antibacterial cephalosporins had the highest DDD, followed by penicillins and imidazole derivatives. Similar data have been observed in the African region (World Health Organization, 2019a). In other regions, some differences have been noted, such as tetracyclines that had the highest DDD per 1,000 outpatients per day and aminoglycosides that had the highest DDD per 1,000 admissions per day (World Health Organization, 2019a).

In Mauritius, the DDD per 1,000 admissions in the regional hospitals was also higher than in the specialized hospitals. The reason may be due to the inclusion of Brown Sequard Mental Hospital, which has a low antibiotic consumption, and Subramanian Eye Hospital, which uses mostly antibiotics in eyedrop form, among the specialized hospitals.

According to the ECDC report on antimicrobial consumption, in 2017, the average consumption of carbapenems in the hospitals of Europe was 0.06 DID and ranged from 0.02 to 0.17 (European Centre for Disease Prevention and Control, 2018). Compared to the European countries, the DID of carbapenem (0.02–0.03) in Mauritius can therefore be considered to be lower. However, looking at the AST data in Mauritius, the proportion of patients infected with K*lebsiella*, *Acinetobact*er species, and *Pseudomonas aeruginosa* species that were sensitive to meropenem was between 80% and 90%, 45% and 47%, and 50% and 67%, respectively. In Europe, the average resistance of *Klebsiella*, *Acinetobacter species*, and *Pseudomonas aeruginosa* to carbapenem was 7.2% (sensitivity of 92.8%), 33.4% (sensitivity 66.6%), and 17.4% (sensitivity 82.6%), respectively (European Centre for Disease Prevention and Control, 2017). It is important to note that while interpreting that data in Europe, all the above-mentioned invasive isolates were included, while for this study in Mauritius, only data on bacteria in blood samples were collected and analyzed.

Another alarming finding is that the proportion of isolates susceptible to meropenem, ceftriaxone, and ciprofloxacin was seen to decrease from 2015 to 2023. The proportion of samples of *E. coli*, *Klebsiella pneumoniae*, *Acinetobacter baumannii*, and *Pseudomonas aeruginosa* sensitive to meropenem decreased from 2015 to 2023 from 98% to 94%, 83% to 53%, 45% to 28%, and 63% to 47%, respectively. From 2015 to 2023, the proportion of *E. coli* and *K. pneumoniae* isolates sensitive to ceftriaxone decreased from 55% to 39% and from 37% to 22%, respectively. This increase in resistance of *E. coli and K. pneumoniae* to carbapenems has been observed, on average, in the countries of Europe as well according to the ECDC report 2021. This increase in resistance to the last-resort antibiotics is alarming as this decreases the treatment options available for sick patients who may go into multi-organ failure, develop complications, and die. The situation is hence critical, and it is urgent for the authorities to take urgent actions to address this issue.

Moreover, the results showed that more than 50% of the meropenem-resistant *Klebsiella* and *Acinetobacter* species were isolated in the intensive care units of the regional hospitals of Mauritius. Another study done in 2020 in the ICU of one hospital showed this high prevalence as well (NuckChady and Boolaky, 2020). It was observed that those carbapenem-resistant organisms led to an increase in antibiotic use and prolonged hospital stay (NuckChady and Boolaky, 2020). However, although more than 50% of the carbapenem-resistant strains were identified in the ICU, it is important to take appropriate measures as reinforcement of the infection and prevention control in other wards as well.

A study done in Reunion Island shows the CRE to be high in patients coming from Mauritius for medical treatment (Gay et al., 2020). The high CRE detected can be due to the severity of the patients’ illness that were treated in Mauritius, which required carbapenems. Mauritius, having a free healthcare system and access to carbapenems, can provide the necessary medications to the patients. Another reason is the high flux of tourists and patients going abroad and coming to the island of Mauritius (population of approximately 1.2 million) for holidays or medical treatment each year (European Centre for Disease Prevention and Control, 2017; Ministry of Tourism, 2019). In 2019, there were 1.8 million arrivals, among whom 1.4 were non-Mauritian citizens (Statistics Mauritius, 2019). These persons might be asymptomatic carriers of CRE, and they can, in turn, spread the resistant bacteria in their country (Frost et al., 2019; Ruppé et al., 2014; Ruppé et al., 2015). Since the consumption and use of carbapenems is found to be low in the country, it is vital to carry out surveys and studies to determine the cause of this high CRE rate in the hospitals of Mauritius—for example, studies should be carried out on the effect of travel on the microbiome in persons coming from abroad.

The proportion of MDR strains in blood samples in Mauritius was compared with that of the European Centre for Disease Prevention and Control report 2017 (ECDC) as well as that of India (2014) and Malawi (2016) (European Centre for Disease Prevention and Control, 2017; Musicha et al., 2017; Nagvekar et al., 2020). The proportion of MDR *Klebsiella pneumoniae* was found to be 50%–60% of the isolated bacteria in Mauritius against 15.5% in Europe, 44% in India, and 92% in Malawi. The proportion of MDR *Acinetobacter* species was found to be 50%–60% of the isolated bacteria in Mauritius against 43.2% in Europe and 89% in India. The proportion of MDR *E. coli* was also 25%–41% in Mauritius, 7.3% in Europe, 9% in India, and 69% in Malawi. Concerning MDR *Pseudomonas aeruginosa* isolates, the proportion was 25%–41% in Mauritius, 4% in Europe, and 30% in Malawi (European Centre for Disease Prevention and Control, 2017; Musicha et al., 2017; Nagvekar et al., 2020). The MDR rates in Mauritius are comparable to that of India and Malawi but higher compared to Europe. The choice of samples in Europe might explain the above-mentioned data as the samples collected in the ECDC report included cerebrospinal fluid along with blood, while for this study in Mauritius, only blood samples were included (European Centre for Disease Prevention and Control, 2017).

However, it is vital to note that from 2015 to 2023, the proportion of MDR *Acinetobacter baumannii* and *Pseudomonas aeruginosa* has further increased from 58% to 74% and from 33% to 45%, respectively. Some reasons explaining this increasing MDR and high prevalence of resistance can be poor infection prevention control in the hospitals, misuse of antibiotics, non-adherence to the dose of antibiotics as prescribed by the treating doctor, importation of animals with resistant bacteria, the high flux of tourists in the country, or the choice of patients in whom blood culture was done. Additional studies should be done to follow the trend of MDR bacteria and determine the causes of this increasing rate.

The first limitation of the study is the lack of information on the amount of antibiotics expired, thrown away, and not used by the patients or healthcare staff. While the pharmacists and dispensers are of the opinion that the amount expired and thrown away is negligible, we have no data on the total amount of purchased antibiotics by patients and those remaining at the end of the year. Moreover, some interviews with patients in health centers revealed that the antibiotics were taken only for a few days until the recovery of the patients. The whole course was not taken. These information are vital when interpreting the data on antibiotic consumption.

Another limitation is the inclusion of resistance data for only four pathogens isolated in blood culture in the public hospitals in this study. The reason is data collection is long and tedious as data on resistance had to be retrieved manually from the register. The resistance data also included only blood samples in the public sector. Having antibiotic resistance data from various samples and in the private sector would be interesting, especially with the higher WATCH group of antibiotic consumption in the private sector. In 2023, data on ABR only included January to May 2023, while data in 2015, 2016, 2017, and 2021 was from January to December. While the results in 2023 have to be interpreted with caution, it is important to note that no seasonal trend in ABR was observed during the years 2015 to 2021.

A last limitation is the lack of data in the animal sector concerning antibiotic consumption by species and live weight (PCU) as well as the prevalence of antibiotic resistance.

Several studies have acknowledged the link between antibiotic consumption and resistance, while others are still investigating the different factors causing and spreading antibiotic resistance across the world (Bakhit et al., 2018; Frost et al., 2019; Goosens, 2009; Ruppé et al., 2014; Tamminen et al., 2011; Meyer et al., 2013; Bell et al., 2014; Oz et al., 2014; Ruppé et al., 2015; Baditoiu et al., 2017; NuckChady and Boolaky, 2020).

Just as with the COVID-19 outbreak, no one is safe until everyone is safe. Indeed drug-resistant organisms do not respect national borders; they can affect people of any race, gender, or color as well as animals. Addressing this issue requires global cooperation, and each country should make antimicrobial resistance a top priority in their agenda. In order to understand the burden of antibiotic consumption and resistance, electronic surveillance is essential. Additionally, implementing antibiotic stewardship programs in the human and animal sectors is crucial for responsible antibiotic use and prescription. In the animal sector, a study on farm animals has been undertaken to assess the presence of MDR bacteria in Mauritius (Ministry of Agro-Industry and Food Security, 2022). Moreover, a One Health committee has been established, bringing together the Ministry of Health and Wellness, the Ministry of Agro-Industry, and partners including the SEGA network of the Indian Ocean Commission to address AMR in the country. This committee facilitates information sharing and guides the policy makers in making informed decisions to tackle antibiotic resistance across all relevant sectors.

## 5 Conclusion

This study provides valuable insights that should be taken into consideration when implementing the National Action Plan on AMR in Mauritius. The findings show that the consumption of antibiotics in human medicine is considerably higher than in animals. There is a higher consumption of antibiotics belonging to the WATCH and RESERVE categories in the private healthcare sector, and more urgently, a decrease in the proportion of *Escherichia coli*, *Klebsiella pneumoniae*, *Acinetobacter baumannii*, and *Pseudomonas aeruginos*a susceptible to several WATCH and RESERVE antibiotics was observed from 2015 to 2023. Moreover, the study highlights an increasing trend of MDR strains of the above-mentioned bacteria in the hospitals of Mauritius and a high prevalence of carbapenem-resistant bacteria across various hospital wards, not just in ICUs. Hence, it is imperative to establish a national surveillance system to monitor antibiotic consumption and resistance using a One Health approach. This comprehensive approach is key to understand the burden of AMR in the country, enabling policymakers to implement effective strategies to address this growing threat of AMR in Mauritius.

## Data availability statement

The raw data supporting the conclusions of this article will be made available by the authors, without undue reservation.

## Ethics statement

The studies involving human participants were reviewed and approved by Ethics Committee of the Ministry of Health and Wellness, Mauritius. Written informed consent from the participants’ legal guardian/next of kin was not required to participate in this study in accordance with the national legislation and the institutional requirements. Ethical review and approval was not required for the study on animals in accordance with the local legislation and institutional requirements.

## Author contributions

LV-M collected, analyzed and interpreted the data. She conceived the original paper and provided a rough draft. EC, MI, and HR-A interpreted the data, checked the paper for consistency, corrected the language and checked the references for consistency and accuracy. All authors contributed to the article and approved the submitted version.
